# When good intentions go wrong: medication errors associated with adverse events in veterinary practice

**DOI:** 10.1186/s13028-026-00885-2

**Published:** 2026-10-09

**Authors:** Anni Järvinen, Annika Kiiski, Marja Raekallio, Anna-Riia Holmström

**Affiliations:** 1 Division of Pharmacology and Pharmacotherapy, Faculty of Pharmacy, University of Helsinki, Viikinkaari 5 E, Helsinki, 00014 Finland; 2 HUS Pharmacy, University of Helsinki and Helsinki University Hospital, Stenbäckinkatu 9, Helsinki, 00290 Finland; 3 Department of Equine and Small Animal Medicine, Faculty of Veterinary Medicine, University of Helsinki, Agnes Sjöbergin katu 2, Helsinki, 00014 Finland

**Keywords:** Adverse event, Critical incident, Medication error, Medication safety, Veterinary medicine

## Abstract

**Background:**

Medication safety is a central component of patient safety. Although some studies on medication errors have been conducted in veterinary medicine, data on the prevalence and severity of errors remain scarce. This study investigated veterinary medication errors using Finnish authorative adverse event data about claimed medical malpractice. The study comprised a retrospective quantitative document analysis of 256 claims cases reviewed by the Assessment Board for Damages in Veterinary Practice between 2014 and 2023.

**Results:**

A total of 87 cases met the inclusion criteria, in which 148 medication errors were identified. Medication errors occurred most frequently in dogs (*n* = 57/148, 65.5%) and horses (*n* = 12/148, 13.8%). The most common error type was omission of medication administration (*n* = 25/148, 16.9%). Fatal outcomes occurred in 21.8% of cases (*n* = 19/87), and an equal proportion (*n* = 19/87, 21.8%) involved severe adverse events resulting in injury or other life-threatening conditions. In total, 223 medications were involved, most commonly systemic antibacterials (*n* = 33/223, 14.8%), anti-inflammatory medications (*n* = 32/223, 14.3%), and anesthetics (*n* = 19/223, 8.5%). The oral route of administration was most frequent (*n* = 73/223, 32.7%). Most medication errors occurred during the decision-making phase (*n* = 85/148, 57.4%), and errors occurred most commonly in veterinary clinics and animal hospitals.

**Conclusions:**

Targeted measures to strengthen the decision‑making phase of veterinary medication process are warranted. The most frequent medication groups associated with errors were NSAIDs, systemic antibacterial agents, and sedative and anesthetic medications. Further research is needed to better identify and prevent medication errors and to support the development of effective risk management strategies in veterinary medicine.

**Supplementary Information:**

The online version contains supplementary material available at https://doi.org/10.1186/s13028-026-00885-2.

## Introduction

Medication safety refers to the safe use of medications through practices that prevent medication-related harm and promote optimal therapeutic outcomes [1, 2]. Ensuring medication safety has become a critical aspect of patient safety in both human and veterinary healthcare [2, 3]. In human medicine, medication errors are relatively common, with reported incidence rates in hospital settings ranging from 0.38% to 5% [4–7] However, the proportion of serious medication errors remains uncertain due to inconsistent reporting practices [8], and definition of severity of outcomes vary because no universally accepted international standards exist [9].

In veterinary medicine, comprehensive data on the prevalence and severity of medication errors is limited. Existing studies from Europe and the United States suggest that up to 42% of adverse events, defined as incidents in which a patient is harmed, and 15% of medication errors result in harm to animals, with 8% leading to permanent injury or death [3, 10]. Medication safety is becoming increasingly important as veterinary care grows more complex. Advances in diagnostics and treatment options, the involvement of larger multidisciplinary veterinary teams, and increasing awareness of critical incidents all contribute to the need for safe medication management processes. In addition, pet ownership remains widespread, with 49% of European households owning a pet in 2024 [11]. As pets are increasingly regarded as family members, owners are often willing to invest in more advanced treatments [12]. Consequently, evaluating medication management processes and identifying related risks is essential for ensuring safe veterinary practice.

A widely used systems approach to patient safety, grounded in James Reason’s model of human error, conceptualizes errors as inherent to human activity, emphasizing that they are an unavoidable but preventable part of processes involving human action, including veterinary medicine [13]. Systems that rely solely on flawless individual performance are insufficient to prevent errors. Instead, understanding the nature and contributing factors of medication errors can support the development of system-based organizational strategies that effectively prevent such incidents [14]. Despite this need, research on veterinary medication errors remains limited, and only few prior studies have specifically focused on medication errors and their characteristics. To address the present gap, this study aimed to characterize medication errors involved in veterinary adverse events, where the owners of the animals suspected medical malpractice, by identifying their types, associated medications and animal species, level of harm caused, and the phases of the medication management and use process in which they occurred.

## Material and methods

### Study design and context

The study was a retrospective document analysis of claims for damages related to adverse events involving animals, where the owner of the animal suspected medical malpractice by a veterinarian. The study was conducted in the context of Finland, where such claims are assessed by the Assessment Board for Damages in Veterinary Practice (ABDVP) operating under the Finnish Food Authority. The ABDVP assesses and issues statements regarding the actions taken by veterinarians in treating animals or other procedures related to animal care [15]. An application for an expert evaluation may be submitted by the animal owner, the veterinarian, or the veterinarian’s employer. However, in the vast majority of cases, the application is submitted by the animal owner. The application must be submitted using the ABDVP application form. Upon receipt of the application, ABDVP obtains the expert opinions and other relevant documentation. A rapporteur employed by the Finnish Food Authority prepares the case for the Board’s consideration and decision.

The research material consisted of cases reviewed by the ABDVP between 2014 and 2023. The board evaluated whether the veterinarian responsible for the treatment committed a medical error and whether there was a causal link between the error and the resulting harm.

### Data collection and definitions

In the present study, the term “case” refers to a request for a statement submitted to the ABDVP, whereas “error” indicates a medication-related incident affecting an animal; multiple errors could occur within a single case. The study included all cases involving a medication error that were processed by the ABDVP during the study period. If a single case involved multiple medication errors, each error was treated as a separate event. For example, if a veterinarian prescribed an incorrect dose of a pain medication, this was recorded as a single medication error, even if the owner administered the medication multiple times. If the medication was administered to the same animal at an excessive dose and via an incorrect route of administration, these were recorded as two separate errors. If the reported error was premature discharge following a procedure performed under anaesthesia, all medications used during the anaesthetic procedure were recorded as being associated with that one medication error. Premature discharge was classified as inadequate medication monitoring. In this study, monitoring of a patient’s response to administered medications, including assessment of recovery from anaesthesia, was considered part of the medication process, consistent with medication error frameworks that include monitoring of medication effects as a component of the medication-use process (e.g [16, 17]), . Consequently, incidents involving discharge before adequate recovery from anaesthetic medications were categorized as medication-related events within the study classification framework.

A medication was defined as any veterinary medicinal product as defined in Regulation (EU) 2019/6, as well as rehydration solutions, trace elements, vitamins, glucose preparations, and oxygen used in veterinary care. Blood products were excluded from the study in accordance with the regulation.

The level of animal harm associated with each medication error was assessed based on the expert opinions of ABDVP specialists, including their assessment of whether the error was likely to have resulted in, for example, an injury or prolonged hospitalization. When the ABDVP specialists did not provide an assessment of the harm attributable to an individual medication error, the case was discussed within the research group until a consensus was reached. “Unclear” was included as a possible harm category when there was insufficient information to determine whether the medication error had contributed to the observed outcome.

**Fig. 1:**
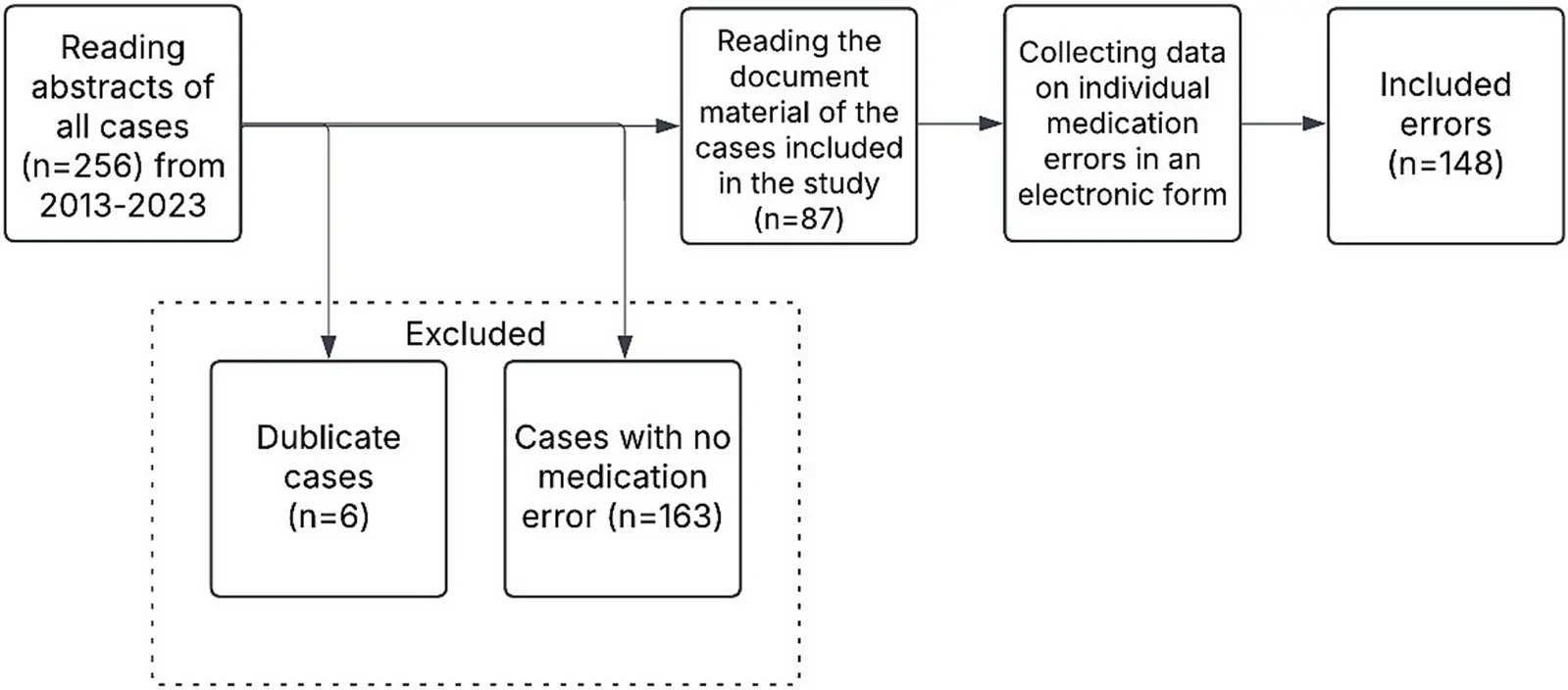
The process of compiling the research data. The process how the final research dataset was compiled from claims cases reviewed by Assessment Board for Damages in Veterinary Practice between 2014 and 2023

The data were stored in paper format at the ABDVP and were collected and categorized by a researcher (AJ). In the first phase, the researcher determined whether the case involved a medication error, primarily based on an abstract of the case. The process of compiling the final research dataset is illustrated in Fig. 1.

### The data collection instrument

Data was collected through an electronic form adapted from an earlier study [18]. The form was further modified during the data collection process to better align with the information gathered. The final applied form is presented in additional file 1.

### Analysis

A descriptive quantitative analysis reporting frequencies (n) and percentages (%) was performed. The collected data were analyzed using cross-tabulation. To ensure the reliability of data categorization, another researcher (AK) independently assessed 12 randomly selected cases (13.8%, n *=* 12/87). Cohen’s kappa was calculated to evaluate inter-rater reliability in classifying medication error types. The James Reason’s systems approach to medication risk management guided the interpretation of the research findings [13].

### Ethics

The study was conducted in accordance with the ethical principles of the Finnish National Board on Research Integrity [19]. Approval for the use of the research material was granted by the ABDVP.

## Results

A total of 87 cases involving at least one medication error were included in the study, representing 34.8% of all cases (*n* = 250) reviewed by the ABDVP between 2014 and 2023. As each medication error within a case was treated as a separate incident, the final dataset comprised 148 individual errors (Fig. 1).

### Background information

Dogs (*n* = 57, 65.5%) and horses (*n* = 12, 13.8%) were the most frequently reported species among the cases (*n* = 87). Cats (*n* = 9/87, 10.3%) and other mammalian species (*n* = 9/87, 10.3%) were also represented. Of all animals, 56.3% (*n* = 49/87) were female, 40.2% (*n* = 35/87) male, and in three cases (3.4%), the sex was not reported.

### Occurrence sites of medication errors

Most errors occurred in veterinary clinics or animal hospitals (Table 1). Of all errors (*n* = 148), the majority (62.8%, *n* = 93) occurred in the private sector, whereas 37.2% (*n* = 55) in public sector. In one case the sector could not be determined.

**Table 1:** Occurrence sites of medication errors (*n* = 148)

Animal species/ Occurrence site	Dogs, *n* (%)	Horses, *n* (%)	Cats, *n* (%)	Other, *n* (%)	Total, *n* (%)
Veterinary hospital	23 (15.5%)	2 (1.4%)	6 (4.1%)	-	31 (21%)
Veterinary clinic	73 (49.3%)	3 (2.0%)	17 (11.5%)	1 (0.7%)	94 (64%)
Field practice	-	10 (6.8%)	-	11 (7.4%)	21 (14%)
Phone communication	1 (0.7%)	-	-	1 (0.7%)	2 (1%)

### Error types and level of animal harm

The types of medication errors are presented in Table 2 and described in more detailed in additional file 2. The most common error type was omission of medication administration, accounting for 16.9% (n *=* 25/148) of all errors. The second most common type was inadequate monitoring of medication by the veterinarian (n *=* 20/148, 13.5%), often involving cases where a sedated or anesthetized animal was discharged prematurely according to the ABDVP statement or while still markedly sedated. This category also includes deficiencies related to the monitoring of medications administered at home. Fatal harm occurred for 21.8% of the animals (*n* = 19/87), and an equal proportion (*n* = 19/87, 21.8%) had serious harm accounting for injury, prolonged hospitalization, or other life-threatening conditions. The outcomes of individual error types are presented in additional file 3.

**Table 2:** The most common types of medication errors (*n* = 148)

Animal species/ Type of error	Dogs, *n* (%)	Horses, *n* (%)	Cats, *n* (%)	Other, *n* (%)	Total, *n* (%)
Omission of medication administration	16 (10.8%)	2 (1.6%)	5 (3.4%)	2 (1.6%)	25 (16.9%)
Inadequate monitoring of medication	15 (10.1%)	-	3 (2.0%)	2 (1.6%)	20 (13.5%)
Failure to consider contraindications	11 (7.4%)	-	5 (3.4%)	-	16 (10.8%)
Improper dose	9 (6.1%)	3 (2.0%)	1 (0.7%)	3 (2.0%)	16 (10.8%)
Incomplete background information	11 (7.4%)	-	-	-	11 (7.4%)
Wrong medication	3 (2.0%)	2 (1.6%)	3 (2.0%)	1 (0.7%)	9 (6.1%)
Other	32 (21.6%)	8 (5.4%)	6 (4.1%)	5 (3.4%)	51 (34.5%)

### Medications involved in errors

A total of 223 medications were identified in the reported errors (*n* = 148). Based on the ATC classification, the highest number of errors were observed in antibacterials for systemic use (*n* = 33/223, 14.8%), anti-inflammatory and antirheumatic products (*n* = 32/223, 14.3%), and anesthetics (*n* = 19/223, 8.5%). A complete list of medicines involved is provided in additional file 4. Meloxicam was the most commonly reported individual drug (Table 3). The most frequently reported administration routes were oral and intravenous administration (Table 4).

**Table 3:** The most frequently involved medications (*n* = 223) in medication errors (*n* = 148)

Medication	*n* (%)
Meloxicam	15 (6.7%)
Amoxicillin/clavulanic acid	11 (4.9%)
(Dex)medetomidine	9 (4.0%)
Carprofen	8 (3.6%)
Propofol	7 (3.1%)
Butorphanol	6 (2.7%)
Enrofloxacin	6 (2.7%)
Oxytocin	5 (2.2%)
Metamizole combinating drug	5 (2.2%)

One medication error could include multiple medications (e.g., wrong dosing of multiple medications at the same time)

**Table 4:** Administration routes of medications (*n* = 223) involved in medication errors (*n* = 148)

Administration route	*n* (%)
Oral	73 (33%)
Intravenous	56 (25%)
Intramuscular	25 (11%)
Subcutaneous	9 (4%)
Inhalation	9 (4%)
Otic	8 (4%)
Other	7 (3%)
Ocular	5 (2%)
Information not available	31 (14%)

One medication error could include multiple medications with different administration routes

### Stage of the medication management and use process

Most medication errors occurred during the decision-making and monitoring phases of treatment (Table 5).

**Table 5:** Occurrence of medication errors (*n* = 148) at different stages of the medication management and use process

Stage of medication process	*n* (%)
Decision on treatment	85 (57.4%)
Monitoring treatment	38 (25.6%)
Administering medication	10 (6.8%)
Prescribing medication	4 (2.7%)
Providing medication counseling	4 (2.7%)
The use of medication at home	3 (2.0%)
Other	4 (2.7%)

## Discussion

This study described medication errors in animals using national authoritative data on claims of adverse events. The most common type of error was omission of medication, while dosage errors accounted for approximately one-tenth of the cases. Previous research has identified that the most common medication errors in veterinary medicine involve wrong drug, wrong dosage, or incorrect dosing frequency [10, 20]. The discrepancy between our findings and those reported in earlier studies may be explained by the fact that omission errors are difficult to detect and are rarely captured by traditional research methodologies, such as medication error reporting systems or medical chart review [21]. The characteristics of our data may also explain the lower prevalence of dosage errors, which may also go unnoticed by pet owners. Furthermore, studies in human healthcare have demonstrated that medication errors are classified differently across investigations [16, 22]. A similar situation likely applies in veterinary contexts, thereby complicating comparisons between existing studies.

The most medication errors occurred during the decision-making phase of treatment, which aligns with previous findings [23], where more than half of errors occurred during the treatment decision process in large animals. The WHO systematic review [7] also found that medication errors in human healthcare most often arise during the ordering or prescribing phase. In the veterinary context, this may manifest when dosing instructions are unclear or ambiguous, leading pet owners to administer the medication incorrectly. Errors during the ordering or prescribing phase may also occur as a result of prescriber-related human error, resulting in, for example, incorrect dosing [24]. Clinical decision support systems, such as medication dose range calculation for different species and other IT-based solutions could serve as protective measures against such medication errors and enhance patient safety [25, 26].

Medication errors occurred most frequently in dogs, consistent with previous findings [24]. Similarly, previous research has demonstrated that such errors were twice as common in dogs as in cats [10]. These findings may be attributed to the popularity of dogs as pets and the complexity of medical procedures performed on them, which likely influences complaint rates. Furthermore, dog owners tend to use veterinary services more frequently and invest more in their pets’ care compared to cat owners, including in life-saving procedures [27, 28]. Conversely, a significantly higher proportion of cats were harmed by incidents compared to dogs [10]. Further research involving larger animal populations and diverse data sources is needed to better identify species-specific risks and inform protective interventions.

The medication classes most frequently associated with medication errors were anti-inflammatory and antirheumatic products and antibacterials for systemic use. Similarly, these classes were among the most commonly involved in errors in large animal teaching hospitals the United States [23]. A study conducted in Slovenia identified meloxicam and carprofen as the most commonly prescribed NSAIDs for cats and dogs [29], illustrating the widespread use of these substances in veterinary practice. The extensive use of these medications may partly explain their high prevalence in our dataset, as frequently administered medications are probably more likely to be involved in reported medication errors.

Anesthetic agents, including sedatives and opioids, were commonly observed in our study. Anesthesia related errors often result in severe incidents and even death in dogs and cats [10]. Due to the characteristics of our dataset and its focus on veterinary clinics and animal hospitals, anesthetic agents may be overrepresented, possibly emphasizing their role and the frequency of intravenous administration errors, both processes considered high-risk in human hospitals [30]. Anesthesia comprises a challenging and high-risk aspect of medication management, despite advancements that have improved safety in veterinary medicine [31–33]. The estimated mortality rate associated with general anesthesia in cats is around 0.11% and in dogs approximately 0.05% [34]. Among dogs undergoing at least one GA/sedation event, the risk of GA/sedative-related death was approximately 0.10–0.14% [32]. This risk was lower among dogs undergoing routine or elective procedures. Older age, certain breeds, poorer health status at the time of GA/sedation, and greater procedural urgency were associated with an increased risk of GA/sedative-related death. For horses undergoing general anesthesia, the overall deathrate is 1.2% [35]. However, data on veterinary medication errors during sedation and anesthesia are limited and often based on voluntary reporting systems which represent only a fraction of true error incidence [36–38]. Potential safeguards for anesthesia and other medication management and use process stages include effective interdisciplinary collaboration and communication, use of checklists for anaesthesia, clear labeling of drugs and syringes, standardized arrangement of medications, and verification procedures; all of which have been shown to reduce anesthesia-related errors by staff [31, 39, 40]. For iv-administration, medication safety in human healthcare has been improved through pharmacist- or industry-prepared prefilled syringes and anesthesia medication templates [41]. Collectively, these interventions reduce dosing variability and enhance medication safety. However, many recommendations established for human medicine may be difficult to implement in veterinary medicine owing to the considerable variation in patient size and species. Moreover, implementing comparable systemic safeguards in veterinary medicine would require substantial resources.

In our study, one of the most common types of medication-related incidents was inadequate treatment follow-up, frequently associated with sedation and general anesthesia. This category was also associated with fatal outcomes and is largely reported as a key high-risk phase of the human medication management and use process [7]. Based on these findings, implementing a standardized protocol to ensure sufficient post-anesthesia and sedation monitoring in veterinary care would be advisable. However, since the patient status and the severity of the procedure varies, no simple, generally applicable instructions can be given.

Moreover, the implementation of guidelines similar to current human care recommendations could help standardize veterinary practice and provide reliable information, especially when veterinarians work with unfamiliar species or under time pressure or fatigue. For example, the Finnish Food Authority has given antimicrobial recommendations for the most significant infectious diseases in animals [42]. Although several consensus statements have been published in peer reviewed journals, producing and regularly updating such statements even for the most common conditions across major domestic animal species would require an excessive investment from the veterinary professional community.

Overall, strengthening professional competencies in system-based medication risk management could improve preventive error management and medication safety in veterinary practice. Pharmacist-led programs, ranging from clinic-based medication safety risk identification and management to clinic-based pet owner medication counselling, as well as other inter-professional models that have reduced medication errors in human healthcare, could similarly support veterinary medication safety [43–45]. Incidents and near-miss events discussed during morbidity and mortality meetings were examined in an Australian veterinary teaching hospital, and the greatest number of recommendations for improvement related to the categories “improving communication/record keeping” and “improving staff training” [24]. Therefore, efforts to improve medication safety should include ensuring that veterinary staff receive appropriate training in effective communication and its importance, while also providing adequate opportunities for communication among staff and with pet owners.

Since the data were collected from owner claims about medication errors by veterinarians or veterinary practices, limited information was available about the mistakes of the owners, although they are typically responsible for administering veterinary medications. Therefore, increasing their awareness of risks and safe practices, as well as communication with healthcare professionals, may also represent a key target of improvement [20, 46]. A study of cat owners’ experiences with administering medications at home found that approximately half of respondents reported receiving sometimes or never guidance on medication administration. Half of owners sought information online, highlighting the need for accessible, reliable educational resources from veterinarians [47]. Practical training and medication packaging design can also facilitate correct administration and reduce medication errors.

Collectively, these considerations highlight the need for further research to develop effective system-level preventive safeguards and error reporting practices to improve medication safety in veterinary care.

### Study limitations

This study has several limitations. The nature of the dataset likely overrepresents preventable or potentially preventable incidents. Applications to the ABDVP are predominantly submitted when owners suspect that the veterinarian could or should have acted differently, and not all adverse events prompt formal complaints. Thus, the dataset reflects only a subset of medication-related incidents. Furthermore, certain methodological decisions may have influenced case classification. A potential limitation is the classification of errors occurring during the medication monitoring phase as medication errors. Monitoring-related incidents may be classified differently across studies and classification systems, and some events included in this category could alternatively be considered patient monitoring or clinical management issues rather than medication errors. This may affect the comparability of our findings with those reported in previous research. Also, medication use that deviated from the Summary of Product Characteristics but complied with the European Union regulations [48] was analyzed in the light of practical constraints inherent in veterinary practice, which may have increased the number of cases deemed non-preventable.

The dataset also predominantly highlights errors attributed to veterinarians, as owners are unlikely to report mistakes made by themselves or other actors. As a result, the cases emphasize veterinarian-committed errors leading to severe outcomes. Additionally, case descriptions were often insufficiently detailed to permit analysis of contributing factors, and the limited documentation restricted the depth of the interpretation. Consequently, this study does not provide a comprehensive overview of all veterinary medication errors in Finland, but offers a focused analysis of selected error types and involved medications. Despite the limitations, the findings provide valuable insights into the nature and complexity of medication-related risks in veterinary pharmacotherapy.

## Conclusions

The present study identified medication-related decision-making and inadequate treatment monitoring as critical risk points for medication errors associated with adverse events in veterinary practice. Medication errors occurred most frequently in veterinary clinics and animal hospitals, with the highest-risk medication class being NSAIDs, systemic antibacterial agents, and sedative and anaesthetic medications. Risk management efforts should therefore pioritize these critical stages of the medication management and use process and the associated medications.

To mitigate the identified risks and enhance medication safety in veterinary settings, system-based risk management strategies are needed. Such approaches include development of treatment guidelines, implementation of electronic clinical decision support systems, training programs on medication risk management, and ensuring adequate care resources within clinical environments. In addition, establishing systematic and standardized data collection on veterinary medication errors would enable better safety monitoring and learning at both organizational and national levels. Further research is needed to better identify and prevent medication errors, particularly those associated with severe adverse events, and to support the development of effective, system-based risk management strategies in veterinary medicine.

## Supplementary Information

Below is the link to the electronic supplementary material.

Supplementary Material 1

Supplementary Material 2

Supplementary Material 3

Supplementary Material 4

## Data Availability

Open data sharing is not applicable to this article as the data applied is highly confidential and can be accessed via Finnish Food Authority. However, restrictions apply to the availability of these data, which were used under license for the current study, and consequently, are not publicly available.
