# Supplementary material for: When good intentions go wrong: medication errors associated with adverse events in veterinary practice
Source: Acta Vet Scand. 2026 Oct 9;68:45. doi: 10.1186/s13028-026-00885-2 (PMC13653378; doi:10.1186/s13028-026-00885-2)
Supplement: Supplementary file 1 [file 13028_2026_885_MOESM1_ESM.docx]

When good intentions go wrong

Anni Järvinen, Anna-Riia Holmström, Marja Raekallio, Annika Kiiski

Supplementary material

Additional file 1

The e-form used for data collection.

E-form:

1. Animal Background

a. Select one: The case concerns: a single patient / multiple individuals
b. Select one: Sex: female / male / unknown
c. Select one: Species: dog / cat / horse / cattle / pig / sheep / guinea pig / hamster / chinchilla / other, please specify: _______

2. Location of the Event (Select one)

a. Animal hospital
b. Private veterinary clinic
c. Public veterinary emergency/clinic
d. Field conditions
e. Home
f. By phone
g. Other, specify: _______

3. Sector of the Event (Select one)

a. Public
b. Private
c. Unknown

4. Medication(s) (1–10)

a. Indication: __________
b. Active substance: __________
c. Brand name: __________
d. ATC level 3: __________
e. ATC level 4: __________
f. ATC level 5: __________
g. Pharmaceutical form: __________
h. Route of administration: __________
i. Special license product: yes / no / unknown
j. Incomplete medication documentation: yes / no / unknown
k. Medication likely a significant factor in the animal’s death: yes / no / cannot assess / additional information: _______
l. Off-label use: no off-label use / different species / different indication / different dosage / different route of administration / use despite contraindication / other, please specify: _______

5. Type of error (Select one)

a. Wrong medication
b. Wrong species
c. Wrong patient
d. Wrong dosage
e. Wrong strength
f. Incorrect administration method
g. Incorrect diagnosis
h. Drug interaction
i. Medication omission (by owner)
j. Medication omission (by veterinarian)
k. Missed dose once/few times during treatment
l. Inadequate treatment monitoring (by owner)
m. Inadequate treatment monitoring (by veterinarian)
n. Ignoring contraindication
o. Incorrect or incomplete drug information
p. Wrong timing
q. Incorrect indication
r. Lack of indication
s. Premature discontinuation of treatment (by veterinarian)
t. Premature discontinuation of treatment (by owner)
u. Incomplete background information (from owner)
v. Failure to follow recommendation (by veterinarian)
w. Inadequate medication documentation
x. Other, please specify: __________

6. Harm to the Patient (Select one)

a. No observable harm
b. Minor harm
c. Serious, observable harm
d. Death: natural / euthanized, cause: _______
e. Unclear

7. Stage of Care Where the Error Occurred (Select one)

a. Treatment/medication decision
b. Prescription
c. Interpretation of the prescription
d. Preparation of medication
e. Compounding/manufacturing
f. Dispensing
g. Delivery of medication
h. Administration to the patient
i. Use of the medication
j. Treatment follow-up
k. Provision of medication advice
l. Other, please specify: __________

8. Preventability of the Deviation (Select one)

a. Definitely preventable
b. Possibly preventable
c. Unlikely to be preventable
d. Definitely not preventable

9. Description of the Event: What happened?
