# Supplementary material for: When good intentions go wrong: medication errors associated with adverse events in veterinary practice
Source: Acta Vet Scand. 2026 Oct 9;68:45. doi: 10.1186/s13028-026-00885-2 (PMC13653378; doi:10.1186/s13028-026-00885-2)
Supplement: Supplementary file 3 [file 13028_2026_885_MOESM3_ESM.docx]

When good intentions go wrong

Anni Järvinen, Anna-Riia Holmström, Marja Raekallio, Annika Kiiski

Supplementary material

Additional file 3

The level of animal harm caused by the medication error (n=148).
