# Supplementary material for: When good intentions go wrong: medication errors associated with adverse events in veterinary practice
Source: Acta Vet Scand. 2026 Oct 9;68:45. doi: 10.1186/s13028-026-00885-2 (PMC13653378; doi:10.1186/s13028-026-00885-2)
Supplement: Supplementary file 4 [file 13028_2026_885_MOESM4_ESM.docx]

When good intentions go wrong

Anni Järvinen, Anna-Riia Holmström, Marja Raekallio, Annika Kiiski

Supplementary material

Additional file 4

Drugs involved (n=223) in the medication errors (n=148) by ATC code. “No ATC code” (n=27) refers to non-medicinal products, oxygen, and medications related to cases of omission. This includes situations in which a veterinarian should have prescribed a medication (e.g., for an infection), but the specific drug was not mentioned in the case description. Water and electrolyte solutions were also included in this category.
